# Periodontal Disease: A Contributing Factor to Adverse Outcome in Diabetes

**DOI:** 10.1111/1753-0407.70136

**Published:** 2025-08-03

**Authors:** Edgard El Chaar

**Affiliations:** ^1^ Department of Periodontics University of Pennsylvania, Denta Medicine Philadelphia Pennsylvania USA

## Abstract

Periodontal disease is a prevalent and chronic inflammatory condition increasingly recognized for its systemic implications beyond oral health. While traditionally confined to dentistry, recent evidence reveals strong associations between periodontitis and chronic systemic conditions such as cardiovascular disease, diabetes mellitus, rheumatoid arthritis, inflammatory bowel disease, Alzheimer's disease, and various cancers. Mechanistic studies have identified plausible biological pathways, including systemic dissemination of periodontal pathogens and immune mediators, which can exacerbate distant organ inflammation and dysfunction. Experimental models highlight how oral bacteria influence immune responses, disrupt gut and vascular homeostasis, and contribute to oncogenesis and autoimmunity. Notably, bidirectional relationships, such as those between periodontitis and diabetes, underscore the need for integrated care approaches. Effective periodontal therapy has demonstrated systemic benefits, including improved glycemic control and reduced inflammation. Given the mounting evidence, periodontal disease should be approached as a critical component of systemic health, necessitating interdisciplinary collaboration among healthcare providers to optimize patient outcomes and public health.

Periodontal disease, a prevalent and chronic inflammatory condition of the oral cavity, is increasingly recognized as a significant contributor to diabetes. Despite advances in dental care and awareness, periodontal disease poses a persistent challenge in healthcare. It is primarily caused by the accumulation of bacterial plaque on the teeth, which, if not adequately removed, can extend below the gum line. This subgingival plaque harbors pathogenic bacteria that initiate an immune response, resulting in inflammation and ultimately bone loss around the teeth. While traditionally confined to the realm of dentistry, a growing body of evidence reveals a compelling connection between periodontitis and systemic health, particularly among persons with diabetes [1, 2, 3].

Epidemiological studies have demonstrated associations between periodontal disease and several chronic disorders, including cardiovascular disease, type 2 diabetes mellitus, rheumatoid arthritis, inflammatory bowel disease, Alzheimer's disease, nonalcoholic fatty liver disease, and certain forms of cancer. These correlations warrant a deeper exploration of the potential causative mechanisms underlying this interplay. In the United States, chronic diseases affect a substantial proportion of the population—more so than in many other countries—driven in part by lifestyle‐related factors such as obesity and metabolic syndrome [3, 4, 5, 6, 7].

A recent Wall Street Journal article highlighted the alarming rates of chronic illnesses, including cardiovascular conditions, rheumatoid arthritis, osteoporosis, Alzheimer's disease, and chronic kidney disease. These diseases not only diminish quality of life but also impose an immense financial burden on the healthcare system [8].

As our population continues to age, the challenge intensifies. Older adults are living longer, yet many struggle with maintaining optimal health, often suffering from multiple comorbidities. Poor oral health, including the progression of periodontitis, becomes more prevalent in aging individuals, thereby increasing their vulnerability to systemic complications. Therefore, it is crucial—both from a medical and therapeutic perspective—to determine whether the observed associations between periodontal disease and systemic illnesses are merely correlative or underpinned by genuine causal mechanisms [9].

In recent years, animal model research has provided strong support for the existence of biologically plausible pathways through which periodontal disease may drive or exacerbate systemic pathology. These studies emphasize how inflammation originating in the periodontium can exert effects that extend far beyond the oral cavity.

One of the primary mechanisms involves the systemic dissemination of periodontal bacteria or immune cells that have been activated locally in the gingival tissues. These bacteria or immune mediators can enter the bloodstream or lymphatic system and travel to distant organs, where they may instigate or worsen disease processes. For instance, Kitamoto et al. [10] demonstrated that oral bacteria‐specific T‐helper 17 (Th17) cells, which expand during experimental periodontitis, can migrate to the gut. There, they are reactivated by translocated oral bacteria and contribute to intestinal inflammation, ultimately promoting colitis. This research illustrates a clear mechanistic link between oral microbial dysbiosis and gastrointestinal immune dysregulation.

Similarly, Atarashi et al. [11] were among the first to show that bacteria of oral origin can colonize the gut ectopically, leading to the expansion of colitogenic T cells and exacerbating colitis in genetically susceptible hosts. These findings suggest that the oral cavity may serve as a reservoir for pathogens capable of triggering inflammatory bowel diseases through systemic migration.

The impact of periodontal pathogens is not confined to the gastrointestinal tract. Abed et al. [12] reported that *Fusobacterium nucleatum* , a well‐characterized periodontal pathogen, exhibits a specific tropism for colorectal cancer (CRC) tissues. This bacterium expresses a Gal‐GalNAc‐binding lectin (Fap2), which binds to host polysaccharides highly expressed in CRC tissues, facilitating its accumulation in tumors and potentially contributing to oncogenesis. This striking ability of oral bacteria to migrate and localize in distant tissues underlines the profound implications of periodontal disease on systemic health.

Furthermore, the presence of periodontal bacteria in extra‐oral tissues can provoke a wide array of inflammatory and functional disturbances. For example, endothelial cells lining the vascular system may become dysfunctional upon exposure to inflammatory mediators and microbial byproducts, contributing to the pathogenesis of cardiovascular disease [11]. A study by Ishai et al. [13] utilized clinical imaging to correlate periodontal inflammation with increased metabolic activity in hematopoietic tissues and arterial walls. These findings support a hypothesis derived from experimental models: that the inflammatory adaptation of hematopoietic progenitor cells in the bone marrow may be a shared mechanism linking periodontitis with other chronic inflammatory diseases.

Zhao et al. [14] expanded on this by identifying a mechanism through which periodontitis may influence other bone‐related disorders. Specifically, they demonstrated that periodontal bacteria‐induced elevation of serum interleukin‐6 (IL‐6) can act on bone marrow to promote the expansion and differentiation of osteoclast precursors. These precursors, in turn, migrate to sites of bone resorption and contribute to excessive bone degradation—a mechanism that may link periodontitis to osteoporosis and other skeletal pathologies.

Another significant finding comes from the work of Konig et al. [15] who identified a potential link between *Aggregatibacter actinomycetemcomitans* and rheumatoid arthritis. This periodontal pathogen produces a leukotoxin (LtxA) that can perforate neutrophils, leading to the generation of citrullinated autoantigens—a hallmark of autoimmune responses in rheumatoid arthritis. Notably, anti‐LtxA antibodies were found to be strongly associated with the presence of anti‐citrullinated protein antibodies (ACPAs) in patients with rheumatoid arthritis, suggesting that periodontal infections may initiate or perpetuate autoimmunity in genetically predisposed individuals.

Dominy et al. [16] added further weight to this theory by demonstrating that *Porphyromonas gingivalis* , a keystone pathogen in periodontitis, can invade brain tissue. In their study, they found both clinical and experimental evidence suggesting that *P. gingivalis* contributes to neuroinflammation and plays a role in the pathology of Alzheimer's disease. These insights point toward a disturbing but critical realization: that chronic periodontal infection may not merely coincide with neurodegenerative diseases but actively drive their progression.

Among the most well‐established bidirectional relationships is that between periodontitis and diabetes mellitus. Diabetes increases susceptibility to periodontal disease through mechanisms such as impaired neutrophil function, altered collagen metabolism, and vascular changes. Conversely, periodontal inflammation negatively impacts glycemic control, creating a vicious cycle. Preshaw et al. [17] showed that individuals with diabetes and severe periodontitis had a threefold increased risk of cardiorenal mortality compared to diabetic patients without severe periodontitis. Moreover, effective periodontal treatment has been associated with reductions in HbA1c levels of approximately 0.4%, highlighting the importance of oral health in metabolic regulation.

Supporting this further, D'Aiuto et al. [18] conducted a well‐controlled randomized trial demonstrating that localized periodontal therapy could improve systemic inflammatory markers, glycemic control, and even vascular and renal function in patients with type 2 diabetes. These results affirm the systemic benefits of maintaining periodontal health, not only in preventing disease progression but also in potentially reversing metabolic dysfunctions.

The Global Burden of Disease (GBD) 2021 [19] study highlights the substantial worldwide impact of oral health conditions. Untreated dental caries in permanent teeth remains the most prevalent condition, affecting an estimated 2.24 billion people globally, with an age‐standardized rate of approximately 27 500 per 100 000 population. Severe periodontitis affects over 1 billion individuals, particularly increasing with age and peaking around 60 years, with a prevalence of 12 500 per 100 000. Edentulism, while less prevalent, still impacts roughly 353 million people worldwide, or 4.11% of the global population. Notably, people with diabetes are disproportionately affected by edentulism, with a weighted mean prevalence of 14.0% compared to 7.1% in non‐diabetics, indicating that diabetics are over twice as likely to be edentulous (OR = 2.39, 95% CI [1.73, 3.28]). Furthermore, individuals with diabetes are 40% more likely to have untreated cavities, and the bidirectional relationship between periodontitis and diabetes is well‐established. Periodontitis is considered the sixth complication of diabetes [20]. These findings underscore the urgent need for integrated healthcare strategies that address both oral and systemic health conditions, particularly in populations with chronic diseases such as diabetes.

Furthermore, the US Centers for Disease Control (CDC) [21] in their 2024 annual oral health and diabetes updates stated that adults aged 20 and older with diabetes are approximately 40% more likely to have untreated cavities than those without diabetes, highlighting a critical disparity in oral health outcomes. There is a well‐established association between diabetes and periodontal disease, a leading cause of tooth loss and a condition that can further complicate glycemic control. Despite this, about 60% of U.S. adults with diabetes had a medical visit in the past year but did not see a dentist, indicating a gap in comprehensive healthcare access. Among adults aged 50 and older, those with diabetes are significantly more affected by tooth loss—being 46% more likely to have fewer than 20 teeth and 56% more likely to have severe tooth loss (eight or fewer teeth) compared to their non‐diabetic peers. Many also report difficulty eating due to dental issues, which can further impact nutrition and overall health. On a national scale, annual dental costs for adults with diabetes are $77 higher (in 2017 U.S. dollars), contributing to an estimated $1.9 billion in additional healthcare spending. Moreover, expanding healthcare coverage to include periodontal treatment for diabetic patients could save individuals an estimated $6000 over their lifetime (in 2019 U.S. dollars). These findings underscore the urgent need for integrated medical‐dental care models that address the bidirectional relationship between diabetes and oral health, promoting better outcomes and reducing long‐term costs.

Considering the substantial evidence now available, periodontal disease is more than just a localized oral condition—it is a systemic health concern with far‐reaching implications. Its role in contributing to the onset, exacerbation, or progression of chronic diseases is supported by robust scientific data. Accordingly, the management of periodontitis should not be siloed within dentistry alone. Rather, a comprehensive, multidisciplinary approach is necessary—one that integrates oral health into the broader framework of systemic disease management.

This integration requires collaborative efforts among dentists and physicians. Patients must also be educated about the systemic consequences of poor oral hygiene and encouraged to adopt preventive strategies that include regular dental visits, professional cleanings, and effective at‐home care.

Ultimately, physicians have an ethical responsibility to consider and address the periodontal health of their patients. By recognizing the oral‐systemic link and taking action to monitor and manage periodontal disease, healthcare providers can significantly improve patient outcomes and reduce the overall burden of chronic illness. Such efforts not only enhance individual well‐being but also serve public health interests by reducing healthcare costs and improving the quality of life on a population level.

## Disclosure

The author has nothing to report.

## Conflicts of Interest

The author declares no conflicts of interest.
